# Pupal productivity in rainy and dry seasons: findings from the impact survey of a randomised controlled trial of dengue prevention in Guerrero, Mexico

**DOI:** 10.1186/s12889-017-4294-8

**Published:** 2017-05-30

**Authors:** Abel Jiménez-Alejo, Arcadio Morales-Pérez, Elizabeth Nava-Aguilera, Miguel Flores-Moreno, Sinahí Apreza-Aguilar, Wilhelm Carranza-Alcaraz, Antonio Juan Cortés-Guzmán, Ildefonso Fernández-Salas, Robert J. Ledogar, Anne Cockcroft, Neil Andersson

**Affiliations:** 1 Centro de Investigación de Enfermedades Tropicales (CIET), Universidad Autónoma de Guerrero, Acapulco, Guerrero Mexico; 2 Unidad Académica de Ciencias Químico Biológicas, Universidad Autónoma de Guerrero, Chilpancingo, Guerrero Mexico; 3 Departamento de Prevención y Control de Enfermedades Transmisibles por Vector, Servicios Estatales de Salud Guerrero, Av. Rufo Figueroa 6, Colonia Burócratas, Chilpancingo, Guerrero Mexico; 4 Centro Regional de Investigación en Salud Pública, 19 Poniente Esquina 4ª Norte s/n, C.P30700 Colonia Centro Tapachula, Chiapas Mexico; 5 CIET International, New York, NY USA; 6 Department of Family Medicine, McGill University, Montreal, Canada; 7 CIET Trust, Gaborone, Botswana

**Keywords:** *Aedes aegypti*, Pupae, Entomological index, Dengue

## Abstract

**Background:**

The follow-up survey of a cluster-randomised controlled trial of evidence-based community mobilisation for dengue control in Nicaragua and Mexico included entomological information from the 2012 rainy and dry seasons. We used data from the Mexican arm of the trial to assess the impact of the community action on pupal production of the dengue vector *Aedes aegypti* in both rainy and dry seasons.

**Methods:**

Trained field workers inspected household water containers in 90 clusters and collected any pupae or larvae present for entomological examination. We calculated indices of pupae per person and pupae per household, and traditional entomological indices of container index, household index and Breteau index, and compared these between rainy and dry seasons and between intervention and control clusters, using a cluster t-test to test significance of differences.

**Results:**

In 11,933 houses in the rainy season, we inspected 40,323 containers and found 7070 *Aedes aegypti* pupae. In the dry season, we inspected 43,461 containers and counted 6552 pupae. All pupae and entomological indices were lower in the intervention clusters (IC) than in control clusters (CC) in both the rainy season (RS) and the dry season (DS): pupae per container 0.12 IC and 0.24 CC in RS, and 0.10 IC and 0.20 CC in DS; pupae per household 0.46 IC and 0.82 CC in RS, and 0.41 IC and 0.83 CC in DS; pupae per person 0.11 IC and 0.19 CC in RS, and 0.10 IC and 0.20 CC in DS; household index 16% IC and 21% CC in RS, and 12.1% IC and 17.9% CC in DS; container index 7.5% IC and 11.5% CC in RS, and 4.6% IC and 7.1% CC in DS; Breteau index 27% IC and 36% CC in RS, and 19% IC and 29% CC in DS. All differences between the intervention and control clusters were statistically significant, taking into account clustering.

**Conclusions:**

The trial intervention led to significant decreases in pupal and conventional entomological indices in both rainy and dry seasons.

**Trial registration:**

ISRCTN27581154.

## Background

Vector borne infectious diseases, including dengue, are an important public health problem, linked to poverty, in Latin America and the Caribbean [1]. It is estimated that there are 390 million dengue infections globally each year, 96 million of which produce clinical disease [2]. The main dengue vector is the *Aedes aegypti* mosquito, which generally inhabits urban habitats and breeds in artificial water containers [3], including those used to store water and those that incidentally accumulate water [4, 5]. *Aedes aegypti* is also the vector for other infections of public health importance, including yellow fever, chikungunya, and zika [6–8].

The main strategy for controlling dengue (as well as chikungunya and zika) is to control the vector, and in particular to control its breeding sites in water containers in and around households. Measurements need to be able to reflect the impact of interventions on vector breeding. Pupae indices are the best estimators of dengue transmission risk, because pupa mortality is minimal compared with larvae mortality [9, 10]. Several pupae indices have been described, including: pupae per household; pupae per person; pupae per hectare; and even more specific ones, such as an index of sexual dimorphism focused on the female pupae. Trials of chemical and other interventions for dengue vector control have reported the impact on different pupae indices [11–14].

After the failure of the dengue eradication programme of the 1950s and 1960s the Pan American Health Organization urged countries to focus their efforts on dengue *control* using an integrated approach, giving priority to environmental management (eliminating mosquito breeding opportunities wherever possible and properly covering the remaining containers), with chemical control using larvicides restricted to containers that could not be controlled by any other means and space sprays reserved for emergency situations [15]. Community participation and health promotion were encouraged but in practice the main activity in most countries was a periodic household visit by personnel from the government’s vector control authority to apply the organophosphate temephos to water containers complemented by occasional space spraying to control the adult mosquito. Between 2007 and 2009, three systematic reviews, although limited by the quality of available evidence, pointed to the value of community participation in reducing *Aedes aegypti* vector density [16–18].

In a cluster randomised controlled trial conducted in Mexico and Nicaragua, we showed that community participation based on socialising evidence for participatory action reduced rates of recent dengue infection, rates of self-reported dengue illness, and four entomological indices of the *Aedes aegypti* vector [19]. The main trial analysis compared entomological indices between intervention and control clusters in the final impact survey, which took place in the dry season. An earlier survey in both intervention and control clusters took place in the preceding rainy season. This article reports an analysis of pupae measurements and other entomological indices in the rainy and dry seasons and examines the impact of the trial intervention on *Aedes aegypti* pupal production in both rainy and dry seasons in the Mexican arm of the trial. Data on pupal productivity from the Nicaraguan arm of the trial are reported elsewhere [20].

## Methods

Details of the Camino Verde trial methods are described elsewhere [19]. Briefly, the trial tested the impact of evidence-based community mobilisation for control of *Aedes aegypti* breeding sites as a means to reduce dengue virus infections and clinical disease, in addition to continuing normal prevention efforts, such as application of temephos to household water containers. The trial took place in sites in Managua, Nicaragua, and in 90 representative clusters in the three coastal regions of Guerrero State, Mexico. In Mexico, half the clusters were randomly allocated to receive the intervention, with the remaining clusters acting as controls. The trial impact survey took place in two phases in 2012; the first phase in August–September 2012 was in the rainy season, and the second phase in November–December 2012 was in the dry season. Both phases included an entomological survey of the households in intervention and control clusters.

### Entomological survey

Trained fieldworkers, working in pairs, undertook entomological inspections in the 90 clusters, each of around 130 households, while other fieldworkers undertook household interviews in the same households. The field teams re-visited closed households up to three times. With the consent of the householder and accompanied by a household member, the fieldworkers recorded container types and locations, presence of temephos in the containers and length of time it had been present in them, whether the containers were closed or open, and the presence of larvae and/or pupae in the containers. A container was considered positive if it contained at least one larva or pupa.

The fieldworkers collected all larvae and pupae using plastic pipettes or syringes and strainers made of fine netting on a ring attached to a wooden handle. They placed the larvae and pupae into labelled plastic bags and transported them to the laboratory in thermos flasks. In the laboratory, they were stored at −20 °C while awaiting examination by expert entomologists.

The entomologists used a stereoscopic microscope (Olympus ® CX41) and classified and quantified larvae and pupae according to recognized taxonomic codes [21, 22]. They identified and counted as pupae any exuviae and adult mosquitoes found in the samples. We preserved the larvae and pupae samples in a 70% alcohol solution after examination.

### Statistical analysis

Statistical analysis relied on the open-source software CIETmap [23] which provides an interface with the R statistical programming language. We calculated pupae indices for two groups of containers: water storage containers, and containers which incidentally accumulated water (such as discarded items, flower pots, and tyres). We calculated several different indices:

1. Pupal productivity percentage for different container types, calculated as the total number of pupae in the container type, divided by the total number of pupae in all containers, multiplied by 100.
2. Pupae-per-household index (PHI), calculated as the total number of pupae found, divided by the total number of inspected households.
3. Pupae-per-person index (PPI), calculated as the total number of pupae found, divided by the total population of the inspected households.
4. Household index (HI), calculated as the number of households with any larvae or pupae, divided by the total number of inspected households, multiplied by 100.
5. Container index (CI), calculated as the number of containers with any larvae or pupae, divided by the total number of inspected containers, multiplied by 100.
6. Breteau index (BI), calculated as the number of containers with any larvae or pupae, divided by the total number of inspected households, multiplied by 100.

We compared indices between rainy and dry seasons and between intervention and control sites in each season. For the HI and CI, we compared the proportion of positive households or containers between intervention and control clusters. For the PHI, PPI and BI, we first calculated the proportion of households in each cluster that were above the overall mean value and compared this proportion between intervention and control clusters. We tested the statistical significance of differences between groups using a cluster t-test [24] and we report the mean differences between groups and their cluster-adjusted 95% confidence intervals (95% CI).

## Results

The field teams undertook entomological inspections in 11,933 households during the rainy season, where they inspected 40,323 containers and found 7070 *Aedes aegypti* pupae. During the dry season, they performed entomological inspections in 10,684 households and inspected 43,461 containers, finding 6552 *Aedes aegypti* pupae. We were unable to re-visit 10.5% of households 1249/11,933) covered in the rainy season, mostly because their inhabitants had moved away or because nobody was home at the time of the survey.

### Pupal production in different containers in the two seasons

Some 9.3% (3756/40,323) of the inspected containers in the rainy season were positive for *Aedes aegypti* larvae and/or pupae. Most of these containers were found outside the households (73.8%, 29,758/40,323) and just over half were uncovered at the time of the inspection (53.3%, 21,501/40,323). In the dry season later in the year, the CI was lower: 5.8% (2542/43,461) of inspected containers were positive for *Aedes aegypti* larvae and/or pupae. In the dry season, most of the containers were inside the household (75%, 32,626/43,461) and just over half were uncovered (53.6%, 23,287/43,461).

**Fig. 1:**
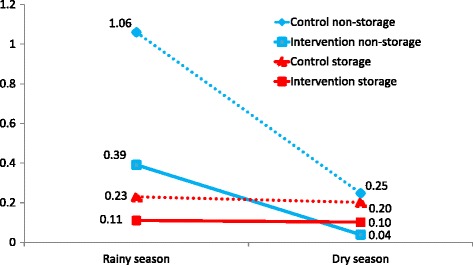
Pupal productivity in containers for water storage and for other purposes, in rainy and dry seasons. Legend: Solid lines represent intervention sites and broken lines represent control sites

As shown in Tables 1 and 2, almost all the containers found and examined were those used for water storage, in both rainy and dry seasons. In the rainy season, the CI and the mean numbers of pupae per container were higher for containers not used for water storage, such as flower pots and tyres. In the dry season, the CI was similar between the different types of container, and the mean numbers of pupae per container were lower in containers not used for water storage (See Fig. 1). Because of the far larger numbers of containers used for water storage, they accounted for almost all the pupal productivity, even in the rainy season.

**Table 1:** Pupal productivity in households by container type during the 2012 rainy season (August–September)

	No. of containers	% of all containers	CI	No. of pupae	Mean pupae /container	% of total pupal productivity
Intervention clusters						
Water storage containers	21,291	97.3	7.0	2365	0.11	91
Pots, tyres etc	596	2.7	25	233	0.39	9
All containers	21,887	100	7.5	2598	0.12	100
Control clusters						
Water storage containers	18,156	98.5	11.2	4175	0.23	93.4
Pots, tyres etc	280	1.5	37.5	297	1.06	6.64
All containers	18,436	100	11.5	4472	0.24	100
All clusters						
Water storage containers	39,447	97.8	8.4	6540	0.17	92.5
Pots, tyres etc	876	2.2	50	530	0.61	7.5
All containers	40,323	100	9.3	7070	0.18	100

*CI* container index (number of containers with any larvae or pupae, divided by the total number of containers in that category × 100)

**Table 2:** Pupal productivity in households by container type during the 2012 dry season (November–December)

	No. of containers	% of all containers	CI	No. of pupae	Mean pupae /container	% of total pupal productivity
Intervention clusters						
Water storage containers	21,707	97.9	4.6	2216	0.10	99.3
Pots, tyres etc	464	2.1	4.5	15	0.04	0.7
All containers	22,171	100	4.6	2231	0.10	100
Control clusters						
Water storage containers	20,887	98.1	7.0	4221	0.20	97.7
Pots, tyres etc	403	1.9	12.2	100	0.25	2.3
All containers	21,290	100	7.1	4321	0.20	100
All clusters						
Water storage containers	42,594	98.0	4.6	6437	0.15	98.2
Pots, tyres etc	867	2.0	12.9	115	0.13	1.8
All containers	43,461	100	5.8	6552	0.15	100

*CI* container index (number of containers with any larvae or pupae, divided by the total number of containers in that category × 100)

### Pupal productivity and pupal density measures in intervention and control clusters

Pupal productivity was consistently lower in intervention clusters compared with control clusters in both rainy and dry seasons (see Tables 1 and 2 and Fig. 1). In the rainy season (Table 1), the mean number of pupae per container was 0.12 in intervention clusters, and 0.24 in control clusters. The proportion of containers with above the *overall* mean number of pupae (0.18) was significantly lower in intervention clusters (0.12) compared with control clusters (0.24) (difference 0.018; cluster adjusted 95% CI 0.009 to 0.028). In the dry season (Table 2), the mean number of pupae per container was 0.10 in intervention clusters and 0.20 in control clusters. The proportion of containers with above the overall mean number of pupae (0.15) was again significantly lower in intervention clusters (0.10) compared with control clusters (0.20) (difference 0.012; cluster adjusted 95% CI 0.002 to 0.023).

**Fig. 2:**
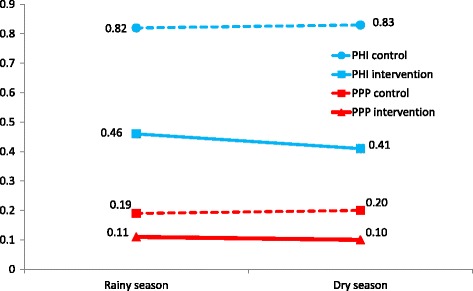
Pupal Household Index (PHI) and Pupae Per Person (PPP), in rainy and dry seasons. Legend: Solid lines represent intervention sites and broken lines represent control sites

The PHI and PPI were consistently lower in intervention clusters than in control clusters in rainy and dry seasons, with little difference in these indices between rainy and dry seasons (Fig. 2). The distribution of the two indices was very skewed, with more than 90% of all households having a value of 0 for the PHI and PPI. Table 3 summarizes the cluster t-tests to test the significance of differences in the indices between intervention and control clusters. The proportions of households with above the overall mean value for either index in intervention clusters were significantly lower than the proportions above the overall mean value in control clusters, in both rainy and dry seasons.

**Table 3:** Cluster t-tests of differences in PHI and PPI between intervention and control clusters in rainy and dry seasons

	Rainy season		Dry season	
PHI				
	Overall mean = 0.58		Overall mean = 0.61	
	Intervention clusters	Control clusters	Intervention clusters	Control clusters
Mean	0.46	0.82	0.41	0.83
Proportion of households above overall mean	0.07	0.11	0.06	0.10
Difference in proportion of households above mean between intervention and control clusters	- 0.04		- 0.04	
Cluster adjusted 95% CI of difference	−0.065 to −0.012		−0.065 to −0.007	
t value	2.961		2.294	
Degrees of freedom	88		88	
*P* value	*p* = 0.004		*p* = 0.024	
PPI				
	Overall mean = 0.17		Overall mean = 0.20	
	Intervention clusters	Control clusters	Intervention clusters	Control clusters
Mean	0.11	0.19	0.10	0.20
Proportion of households above overall mean	0.03	0.05	0.03	0.05
Difference in proportion of households above mean between intervention and control clusters	- 0.02		−0.02	
Cluster adjusted 95% CI of difference	−0.034 to −0.003		−0.042 to −0.006	
t value	2.290		2.500	
Degrees of freedom	88		88	
*P* value	*p* = 0.024		*p* = 0.014	

*PHI* pupae per household index, *PPI* pupae per person index

### Conventional entomological indices in intervention and control clusters

Table 4 shows the values of HI, CI and BI in intervention and control clusters during the rainy and dry seasons. All three indices were lower in the dry season than in the rainy season and they were consistently lower in intervention clusters than in control clusters in both seasons. The cluster t-tests used to test for significance of differences between intervention and control clusters found that the proportion of positives for each index was significantly lower in intervention than control clusters, in both seasons. The tests are summarized in Table 5.

**Table 4:** Entomological indices by season in intervention and control clusters

	Intervention clusters		Control clusters	
Index	Rainy (proportion)	Dry (proportion)	Rainy (proportion)	Dry (proportion)
HI	16% (946/6038)	12.1% (658/5449)	21% (1226/5893)	17.9% (937/5235)
CI	7.5% (1635/21,887)	4.6% (1028/22,171)	11.5% (2121/18,436)	7.1% (1514/21,290)
BI	27% (1635/6038)	19% (1028/5449)	36% (2121/5893)	29% (1514/5235)

*HI* household index, *CI* container index, *BII* Breteau index

**Table 5:** Cluster t-tests of differences in HI, CI and BI between intervention and control clusters in rainy and dry seasons

	Rainy season		Dry season	
HI				
	Intervention clusters	Control clusters	Intervention clusters	Control clusters
Mean HI	0.160	0.210	0.121	0.179
Difference in mean HI between intervention and control clusters	−0.05		−0.06	
Cluster adjusted 95% CI of difference	−0.094 to −0.009		−0.102 to −0.013	
t value	−2.591		−2.439	
Degrees of freedom	88		88	
*P* value	0.011		0.017	
CI				
	Intervention clusters	Control clusters	Intervention clusters	Control clusters
Mean CI	0.075	0.115	0.046	0.071
Difference in mean CI between intervention and control clusters	−0.041		−0.025	
Cluster adjusted 95% CI of difference	−0.062 to −0.020		−0.045 to −0.005	
t value	−3.906		−1.996	
Degrees of freedom	88		88	
*P* value	0.000		0.049	
BI				
	Intervention clusters	Control clusters	Intervention clusters	Control clusters
Mean BI	0.270	0.360	0.190	0.290
Proportion of households above overall mean	0.15	0.20	0.11	0.17
Difference in proportion of households above mean between intervention and control clusters	−0.05		−0.05	
Cluster adjusted 95% CI of difference	−0.093 to −0.009		−0.092 to −0.011	
t value	2.591		2.404	
Degrees of freedom	88		88	
*P* value	0.011		0.018	

*HI* house index, *CI* container index, *BI* Breteau index

## Discussion

The entomological field teams found more containers in and around the households during the dry season than during the rainy season (43,461 vs. 40,323). Despite this, the overall number of pupae found in the households was higher during the rainy season than during the dry season (7070, vs. 6552), and the CI was also higher during the rainy season. These results are similar to those reported by Garelli in Argentina [25] and Maciel de Freitas in Rio de Janeiro (Brazil) [4].

During the rainy season, most inspected containers were found outside the household, whereas most containers were inside the households in the dry season. This reflects the practice of placing containers outside the household during the rainy season in order to collect rainwater, in the face of deficiencies in the water supply. Other authors have reported similar practices [12, 14, 16].

The greatest seasonal variation in pupal productivity occurred in non-storage containers such as pots and tyres, with the mean number of pupae per container falling from 0.61 in the rainy season to 0.13 in the dry season (see Tables 1 and 2). This is in line with findings reported from Morelos in Mexico [26] and from Thailand [27].

In our study, more than 97% of containers identified and examined were those used for water storage, thus these contributed the most to the overall pupal productivity in the households, even during the rainy season when the mean number of pupae per container was higher in the non-storage containers. This contrasts with results from Mérida (Mexico), where non-storage containers were reported to contribute most to the overall pupal productivity rates [5].

There was a clear effect of the trial intervention. The mean numbers of pupae per container were twice as high in control clusters as in intervention clusters, in both rainy and dry seasons. (Tables 1 and 2, and Fig. 1). Similarly, the PHI and PPI indices were twice as high in control as in intervention clusters, in both seasons (Fig. 2). The intervention effects on pupal indices were significant, taking into account clustering.

The intervention impact on HI, CI and BI was also significant in both rainy and dry seasons and of a similar magnitude (a reduction of roughly one third) in both seasons. However, our analysis supports the view that pupae indices are the best estimators of dengue transmission risk for two reasons. First, the values of the pupae-per-household and pupae-per-person indices were quite similar across the rainy and dry seasons (Fig. 2) whereas the traditional indices of HI, CI and BI, whose values were higher in the rainy season (August–September) and fell in the following dry season (November–December), were less stable.

Secondly, traditional *Aedes aegypti* indices only reflect the presence or absence of the vector but pupal indices allow estimation of vector density and transmission risk [9]. Our study confirms the impact of the trial intervention on pupal indices relevant to dengue transmission risk; this helps explain why the intervention was also linked to reduced dengue infections in children (as measured by doubling of specific antibodies) and reduced reported cases of dengue illness [19].

In future studies we may also measure pupal sexual dimorphism, to estimate how many *Aedes aegypti* females emerge [10], and to link this rate to infection transmission levels.

## Conclusion

This analysis of pupal indices and traditional entomological indices in rainy and dry seasons at the end of the Camino Verde trial confirms higher pupal productivity and entomological indices in the rainy season, but with pupae-per-household and pupae-per-person more stable between seasons. It confirms that evidence-based community mobilisation is effective in reducing *Aedes aegypti* infestation and the concomitant risk of transmitting dengue, chikungunya and zika in both the rainy and dry seasons.

## Abbreviations

95% CI: 95% Confidence Intervals
BI: Breteau index
CC: Control clusters
CI: Container index
DS: Dry season
HI: Household Index
IC: Intervention clusters
PHI: Pupae-per-household index
PPI: Pupae-per-person index
RS: Rainy season
